# How to adopt technologies in home care: a mixed methods study on user experiences and change of home care in Finland

**DOI:** 10.1186/s12913-023-10368-z

**Published:** 2023-12-02

**Authors:** Minna Anttila, Juha Koivisto, Minna-Liisa Luoma, Heidi Anttila

**Affiliations:** Finnish Institute for Health and Welfare, Helsinki, Finland

**Keywords:** Ageing, Home care, Technology, Mixed methods, Adoption

## Abstract

**Background:**

There is a need for better adoption of technology to meet the needs of home care professionals, older people, and informal caregivers.

**Methods:**

Mixed methods were used to describe and analyse quantitative and qualitative data in a Finnish governmental programme called KATI. The study was three-fold: it 1) listed what kinds of technologies were piloted and deployed in a national study, 2) provided information from the perspectives of home care professionals about requirements to use technology by using focus-group interviews, and 3) assessed experiences of how the adoption of technology changes work and work processes over time by using repeated surveys. Informants in interviews (*n* = 25) and surveys (*n* = 90) were home care professionals, who also described the perspectives of older people and informal caregivers. The conceptual models framing the study were adapted from the Technology Acceptance Model and DirVA PROVE-IT.

**Results:**

There were 80 technology pilots, in which variety of technologies were followed in home care. Familiarity with, commitment to and understanding of technology benefits were considered to be requirements for the technology to be used. The adoption of technology provided new skills and information about older people’s wellbeing, realisation of treatment and new possibilities in home care. It developed new procedures to focus on older people’s needs and motivated professionals by gained concrete aid. It enabled them to leave out some concrete procedures as unnecessary. On the other hand, there were also pessimistic and negative experiences when technology use provided nothing new or did not change anything.

**Conclusions:**

The adoption of technology is dependent on the technology and its integration into the prevailing service practice. When they both work, it is possible to leave out unnecessary procedures in care, allocate resources and save time. It is possible to be aware of older people’s safety and how they cope at home, find new ways to get involved in older people’s lives, gain insight, and make changes at work. Continuous on-site training, modifications in service practices and communication throughout organisations is needed.

**Supplementary Information:**

The online version contains supplementary material available at 10.1186/s12913-023-10368-z.

## Background

The number of older people is increasing worldwide, resulting in more pressure on health and social care. Professionals working with older people in home care are also getting older in many countries [1, 2]. Similarly, for example in Finland, older people comprise the fastest growing population in terms of internet use [3] and the share of older technology users has grown in the past decade [4]. The benefits of technology use in services for older people come to the fore when technology is adopted to fit into broader social and health care structures. Still, even though technology use has increased in recent years, its use within home care to support older people’s wellbeing needs to be improved [5]. Technology use also raises a range of challenges such as a lack of suitable technologies and existing ones being immature [6]. Therefore, better adoption of technology has been stated [7, 8] to meet the needs of home care professionals, older people, and informal caregivers.

This paper focuses to describe the use of new technologies in home care in Finland. Finland is a country of numerous information systems and technology devices [9] that have difficulties co-operating with each other. Expenditure on long-term home care for older people and people with disabilities has more than doubled between 2000 and 2019 [10]. The experiences of home care professionals in a governmental programme called KATI (‘Smart Ageing and Care at Home’) are studied in this paper. The KATI programme promoted implementation of technology solutions and the adoption of new technology-based practices in home care nationwide in 2021–2023. It also aimed to support the ageing of older people at home, homecare professionals in their work, and home care services in Finland regarding positive welfare in a home care context [11].

In recent years, there have been studies in which healthcare providers’ have used telemedicine in long-term care [12], health information technology has been used by seniors [13], socially assistive robots in older people care [14], and virtual reality among older people [15]. Informal caregivers have also used technology in home health care [16] as well as assistive telecare systems [17]. Technology and device use has benefited older people functioning in daily life, prolonged independent living, and provided access to previously enjoyed activities [18]. However, the users’ needs, capacities, resources, and perceptions to adopt technology vary and had an effect on use.

We now know that, from the home care professionals’ point of view, requirements for technology to be adopted are technology performance, usefulness, and ease of use in daily practice [19]. One way to increase technology adoption is to improve its’ performance expectancy [20]. According to Tan et al. (2021) [12], by offering a wide range of technology services to older people and promoting interprofessional collaboration between acute and long-term care, multidisciplinary professionals could achieve timely on-site management and better quality of care. Brandsma et al. (2020) [20] have also found that technology use can result in information expand, add report functionality, solve log-in problems, and increase speed. However, attitudes of home care professionals [20, 21] can potentially affect how older adults are viewed in relation to technology, and it may influence the adoption of technology-based treatment [21].

From an older person’s point of view, technology must meet certain demands, such as keeping them up with the world [22, 23]. In their systematic review, Kavandi and Jaana (2020) [13] found that the factors that are required from technology do not differ across types of technologies. Factors such as privacy [24–26], security [25], reliability [26], usefulness [24, 26], ease of use [27] and curiosity [24] have been mentioned to positively affect to the needs of older people. Promotion of self-efficacy [25] is of importance in older people’s intention to use technology [25, 27]. On the other hand, technology use has been withdrawn because of lack of time and meaningful use [22]. Further adoption barriers have been unknown price/cost value [13] and the risk of money loss [26]. From the informal caregiver point of view, when they care for older people at home, factors such as information, comprehension, motivation, time, perceived burden, and caregiving competency affect whether they adopt the technology or not [16].

Technology use needs to be harnessed to collect and analyse information to improve processes and health behaviour [28]. Use of technology to increase wellbeing is one of the key goals of the National Programme on Ageing 2030: For an age-competent Finland [29]. As the organisation of public healthcare, social welfare and rescue services are being reformed in Finland, starting from January 2023 responsibility will be transferred from municipalities to wellbeing services counties [30]. With the help of technology, older people could be supported to live at home for as long as possible, considering their needs and their safety. Investing in technology for older people’s care saves costs [31] and creates opportunities for technology companies. More information about new opportunities to detect changes in health issues early, provide good services for older people, increase the staff working time, and improve the flow of information and logistical solutions [29] is urgently needed. Evidence is needed of health benefits as well as whether different technologies can facilitate the work of home care nurses and what factors can positively influence technology use.

## Methods

### Aim

The aim of this paper was to study how to adopt technology to meet the needs of home care professionals (hereinafter professionals), older people and informal caregivers (hereinafter caregivers) in home health care. First, we listed what kinds of technologies were piloted and deployed in the programme. This provided a background as we wanted to explore what requirements there were for professionals, older people and caregivers when they use a variety of technologies in home care. We also wanted to study how the technology affect work and work processes, when technology was adopted in home care and how these changes over time.

### Design

**Fig. 1:**
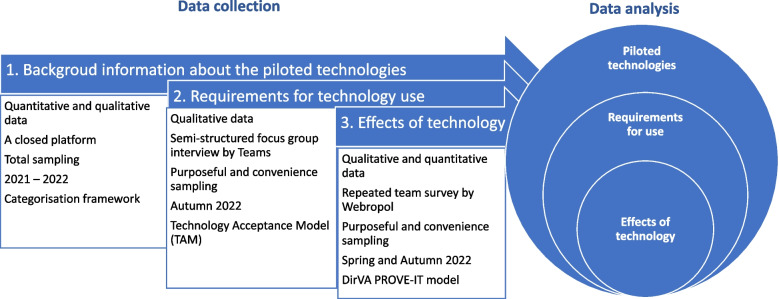
Data integration in the mixed methods study design

A mixed methods research design [32] was adopted to describe the variety of deployed technologies. By adopting such design, we want to provide complementary insights and perceptions that might have been missed if only one research methodology was employed​. We also want to gain deep understanding about the study in focus​ and consider it from multiple viewpoints [33]​. The data from the professionals include their own experiences, as well as the older people and caregiver perspective described by the professionals. Mixed methods sampling, semi-structured focus group interviews, follow-up surveys, and inductive and deductive [34] content-based analysis was used. We also integrate both numbers and narratives, and report findings from qualitative and quantitative strands in discussion [33]. The findings from the data diverge and expand insights and are complementary supplementing each other [35–37] (Fig. 1). Qualitative and quantitative methods together have been found to be the preferred methodology of virtual reality applications in older people [15]. The GRAMMS (Mixed methods studies in health services research) checklist guideline was used in reporting [38].

### Setting

The study setting is the ‘Smart Ageing and Care at Home’ KATI programme, which consists of six regional projects across seven regions in Finland, coordinated by the Finnish Institute for Health and Welfare (THL). THL is an independent state-owned expert and research institute that promotes the welfare, health and safety of the population and operates under the Ministry of Social Affairs and Health (STM). Regional projects had applied for and received funding from the government to be involved in the programme [11]. Based on the regional needs, the projects chose a variety of devices that they piloted or deployed in relation to home care services and at the homes of older people between 04/2021 and 04/2023. The regional projects procured technology, planned pilots or deployments, integrated technologies into information systems, planned ways to fit them into ongoing practices, and considered different practices for customer choices. Moreover, they educated professionals and provided technology solutions to the older people according to needs assessments as part of home care and considered ethical issues for utilising information. Most projects were able to start their pilots in spring 2022.

### Sampling

The total sample used comprised all the projects and their technology deployments across all the KATI projects. Purposeful sampling was used in the regional projects to reach profound and rich information (interviews and surveys) from the professionals regarding the technologies. As an example, as the researchers knew which technologies have been piloted in specific areas, and professionals were encouraged to be informants in relation to this specific technology. Moreover, convenience sampling was used in regional projects to reach the professionals who work in home care and were willing and able to participate in the study. Those eligible were adult professionals (≥ 18 years old) who could speak and read Finnish and were able to give informed consent.

### Questionnaires, interview methods and study subjects

A closed platform was used to collect information about the technologies during the pilots. The contact persons of the regional projects had access to it. They documented the technology name, length of use, and the training time needed by the older people and the professional. Information was collected about errors, support requests and adverse events. Contact persons also documented the number of older people and professionals in the region, the number of older people and professionals who could be potential users of the technology if it were in regular use, and the number of recruited people. As a lack of universal definitions for the classification of the variety of technologies is noticeable [39], we decided to group the technologies into eight categories (Table 1) to represent the results of our study.

**Table 1:** Technology categories and single technologies

Category	Examples of single technologies
A) Remote health measurements (attached to older people)	Measurements of vital signs or weight at home are automatically transferred to care information systems
B) Monitoring technologies	Sleep, activity and nutrition monitoring
C) Technologies installed in the apartment	Home condition measurements: temperature, humidity, lights, safety monitoring system at home
D) Safety-increasing solutions	Wearable safety solution with GPS localisation, medicine-dispensing robot, medicine reminder, monitoring falling
E) Solutions supporting social activity	Virtual peer groups and coffee groups, social robot
F) Solutions supporting rehabilitation	Games activating performance, physical and memory activity solutions, exercise equipment
G) Technologies for care professionals	Electronic homecare optimising system, exoskeletons, virtual homecare or consultation visits, Virtual Reality–based training
H) IoT and integration platforms	Data from devices, applications and services at home are collected on a platform to be further analysed by care professional or AI

Semi-structured focus-group interviews were conducted among voluntary home care professionals across KATI projects. The focus groups were based on our technology categorisation, i.e., those with experiences of technologies within the same category were interviewed at one time. This procedure ensured that information about similar technologies was achieved from several regions while participation did not cause too much of a burden for a single home care site. Interviews were conducted in September 2022. At that time the technologies had been used for some time and the professionals had gained experiences in them. Questions about demographics were asked at the beginning of interview: organisation, education and professional qualification, age, gender, and number of years spent working in home care. The interview guide used in our study included four topics based on the TAM (Technology Acceptance Model) [40], which has been found to be the most applied theoretical framework for older people [15, 41]. The questions were about requirements related to technology use: 1) usefulness, 2) ease of use, 3) attitude towards using technology, and 4) intention to use technology. The interview guide modified from TAM [40] was developed for this study (see Supplementary file 1 for the interview guide). Questions were posed to the professionals from the point of view of themselves, older people and caregivers. Based on the participants’ answers, more detailed questions were asked.

Repeated surveys gathered experiences from voluntary home care teams within a regional KATI project of how their work and work processes change when they adopt technology. Surveys focused on a single technology that was piloted in that area. As an example, a team was asked to respond to a survey as a team in relation to a single piece of technology (e.g., medicine-dispensing robot) that was piloted in their area. This procedure ensured that the teams were able to answer the survey at the most convenient time, and that we were able to receive information about several professionals working at the same site, and about similar technologies from several regions. A baseline survey was conducted between March and May 2022 when the regional projects had just started the technology use. Follow-ups were conducted during October – November 2022 when the technology had been used for some time and the professional had gained experiences in its use, and we were able to get an indication about the long-term experiences of it. Data on the organisation and the number of respondents per team were requested as background information.

The questionnaire used in our study was open-ended and included six topics based on the DirVA PROVE-IT (Prove Outcomes, Value, and Effectiveness of IT in Healthcare) model [28], which is a conceptual model for evaluating digital health technologies and can be perceived as an elaborated CIMO (Context, Interventions, Mechanism and Outcomes) configuration [42, 43]. The questions were about 1) new skills that were provided (a competence or expertise to do a certain task [can do]), 2) information that was produced, 3) new procedures that were formed, 4) previous procedures that were possible to do differently, 5) procedures that can be left as being unnecessary (an ability to act in a specific situation and context or having an idea of what needs to be done [know what to do]), and 6) motivating factors in technology use (will and motivation to do a certain task [want to do]). The questionnaire modified from DirVA PROVE-IT [28] was developed for this study (see Supplementary file 2 for the questionnaire). The topics were asked in the survey from the professional point of view and from the older people and caregiver perspective. Teams wrote their responses in a self-reported survey questionnaire.

### Data collection and recruitment

Professionals in regional projects were recruited via contact persons and email. Contact persons forwarded emails and provided professionals with further information within their regions.

The professionals in the focus group interviews were instructed to inform the first author (MA) via email about their participation in the interview. MA was then able to send them a Microsoft Teams invitation and an information letter to a prescheduled interview, in which their specific technology category was in focus. Participation in a Teams meeting was an expression of informed consent. A short introduction round was conducted when participants had their cameras on, they were reminded of ground rules, confidentiality in interviews and anonymity of results [44, 45]. MA, who led the focus group interviews, is a healthcare professional with a doctoral degree and has experience in conducting focus group interviews. Two other people from the KATI programme (HA, EA, SK, KP) took notes and made sure that all the interview topics were covered. The focus groups included a total of 25 professionals in all the six regional projects across seven areas in Finland. They were all female, 13 were practical nurses and 12 had a higher professional qualification. Their ages varied between 21 and 58 years (mean 39.68). They had working experience within their field of between one and 33 years (mean 12.52). In total, all eight focus groups were conducted based on the eight technology categories. The number of professionals varied from one to five in each interview (mean 3.13). The duration of each interview was around one hour.

For the team surveys, health care teams were instructed to use the link in the email that contact persons from the regional projects sent them via email. An email guided them to the Webropol survey. Surveys were sent to all six regional projects, of which one did not participate. The baseline survey was answered by 21 teams, consisting of a total of 90 professionals. The follow-up survey was answered by 19 teams consisting of 70 professionals. The number of professionals answering the baseline survey varied between one and 10 (mean 4.29) per team and in the follow-up between one and eight (mean 3.68) per team.

### Data analysis

Technology pilots were described by using our categorisation framework. Frequencies, percentages, and average numbers were counted. By doing so we wanted to provide orientative information about the technologies in the KATI programme, and more understanding for the interview and survey results.

The focus group interview data produced 81 pages of notes from two people. The topics based on TAM [40] steered the data categorisation about the requirements. Data was inductively categorised and sub-categorised [46] in line with the questions to identify similarities and differences in the responses. Further analysis was conducted as a whole, despite the technology categories. Kavandi and Jaana (2020) [13] have found in their systematic review that the factors that affect technology adoption do not differ across types of technologies.

Baseline team surveys produced 19 pages and follow-up surveys with 18 pages of text. To describe what may lead to changes in technology use in the home care context, the six topics of the DirVA PROVE IT model [28] were used to steer the data categorisation in the baseline analysis. Data was inductively categorised and sub-categorised in line with the topic questions (similarly to focus group interviews). Each topic (e.g., category question) produced three to five more specific sub-categories, which we described qualitatively to illustrate the participants’ voices. We describe the experiences of all three participant groups’ viewpoints, as well as specific experiences of the participant group in question (professional, older people or caregiver).

In the next step, to gain an overall picture and to assess changes in experiences between baseline and follow-up surveys, quantitative descriptive methods [47] were used. Baseline and follow-up survey responses were analysed deductively by assessing their quantity in line with the qualitative sub-categories. If the same sub-category was found in a team response, it was considered to exist. This procedure was done for all the team responses regarding professional, older people, and caregiver experiences to receive quantitative ratings for all the sub-categories. To assess differences between baseline and follow-up surveys, the existing experiences in team responses out of all the team responses were counted and described as frequencies and percentages. The changes in percentages between baseline and follow-up responses were assessed.

## Results

### Description of technologies piloted in the KATI projects

Altogether, there were around 13,500 home care clients (older people) and 4,500 professionals in the KATI projects. The number of technology pilots was 80. As same technologies were piloted in more than one area, 34 unique technologies were tested or deployed. The pilots took about nine months and training took approximately one hour for the older people and 3.5 h for the professionals. Altogether, around 46% of the older people and 75% of the professionals could have been users if the technology was estimated to be in regular use. Examples of errors, support requests and adverse events are as follows: internet connection or data transfer problems, problems with battery loading, medicine dispensing bags jammed or cups fell down, sensors moved or fell down, and devices got stuck or overheated. Information is presented in Table 2.

**Table 2:** Technology pilots and deployments in the national KATI programme during 2021–22

Length of use	Training time per -older people -professionals	Number of potential users among -older people -professionals	Number of older people in technology pilots
**A) Remote health measurement technologies: 10 pilots in four projects**			
**~ 10 months**	~ 40 min	~ 13%	~ 570
	~ 4 h	~ 78%	
**B) Monitoring technologies: 6 pilots in four projects**			
**~ 6 months**	~ 1 h	~ 70%	~ 80
	~ 4.5 h	~ 79%	
**C) Technologies installed in the apartment: 6 pilots in five projects**			
**~ 9 months**	~ 1 h	~ 20%	~ 140
	~ 2 h	~ 54%	
**D) Safety-increasing solutions: 19 pilots in five projects**			
**~ 7 months**	~ 2 h	~ 13%	~ 750
	~ 2 h	~ 84%	
**E) Solutions supporting social activity: 11 pilots in five projects**			
**~ 11 months**	~ 1.5 h	~ 53%	~ 190
	~ 2 h	~ 54%	
**F) Solutions supporting rehabilitation: 3 pilots in two projects**			
**~ 7 months**	~ 0.5 h	~ 70%	~ 30
	~ 1 h	~ 100%	
**G) Technologies for care professionals: 20 pilots in six projects**			
**~ 10 months**	~ 1 h	~ 25%	~ 1200
	~ 3.5 h	~ 75%	
**H) IoT and integration platforms: 5 pilots in three projects**			
**~ 11 months**	N/A	~ 100%	N/A
	~ 8 h	~ 72%	

### Requirements for technology use

#### Perceived usefulness

Familiarity, commitment to use and understanding of technology benefits were considered requirements for technology uses by the professionals, the older people and their caregivers. The professionals and the older people need to know whom to contact for necessities. Older people and caregivers need to gain benefits of technology use, or they had no special requirements to use technology. Professionals need co-operation between professionals, basic technology skills, interest in technology, and professional skills to guide technology use for older people. From the technologies the older people require performance, and professionals and caregivers require functionality.

For technology to be useful, questions about the impacts of technology use were asked of professionals. They described them as follows. Rehabilitation and appropriate care: professionals can provide appropriate care and learn new ways to encounter older people, and older people gain independence and a sense of human dignity. Resource allocation: technology use impacts are money savings, professionals can plan their work, and those who are unable to physically work in home care are still able to continue their working life. Data collection and monitoring: professionals and caregivers can observe impulses and changes in the health of older people. Safety: older people and caregivers know that older people are being cared for. Fellowship: older people socialise with each other and with caregivers.

#### Perceived ease of use

Professionals considered that the technology use has been easy-going and successful. However, both professionals and older people had had problems at the beginning, but they got used to the technology or modified it to fit to their needs. Technology use needed maintenance; professionals needed to check that the technology worked as expected and that they remembered to use it in their daily work. The older people needed to report if they were unable to use it (e.g., hand shaking, misuse by a relative). Professionals and caregivers had had problems with techniques such as program updates and connection problems.

#### Attitudes towards using technology

Positive experiences were mentioned in relation to technology use. There was an accepting attitude towards it, and this attitude changed to be even more positive when the technology was used. User groups were interested in and favourable towards technology use. They also had a trusting relationship in its use if it was automatic and provided safety. Negative experiences were also mentioned. Attitudes towards technology use were suspicious if the older people were not benefiting from its use or they did not have the capacity to use it. Resistance towards use existed as participants did not want anything new, they did not need the information that it provided, or it was too expensive.

#### Intention to use technology

**Fig. 2:**
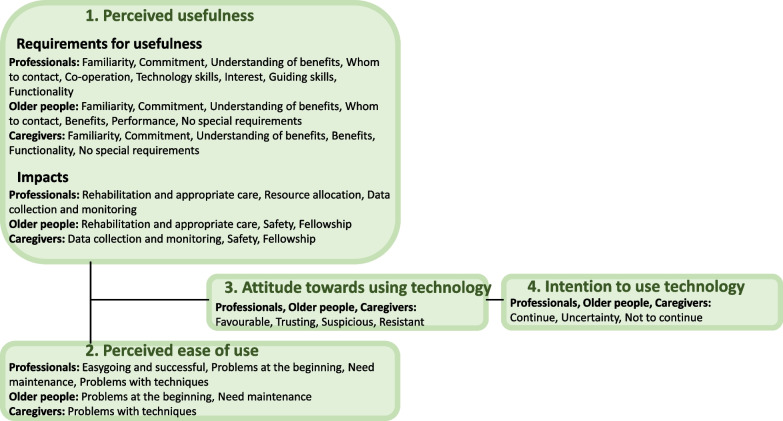
Requirements for technology use (based on Technology Acceptance Model, Davis 1989)

The professionals considered continuing to use the technology as they became more familiar with using it and found new ways to benefit from it. The older people were unwilling to quit using technology, and caregivers were willing to broaden its use. Technology use was considered uncertain in the future. Professionals did not know what equipment and information systems would stay in use after the pilot, if there would be benefits to technology use in daily life, and suspicions about how they could maximally gain benefits from it. The older people were unsure about how to get them later and who would pay for them, and caregivers were unclear about how to obtain them. For some technologies, they were told that their use would not continue, because it was found to be unsuitable, or the equipment was too difficult for older people to use. Figure 2 describes the requirements for technology use.

### Experiences of work and work process change when technology is adopted in home care

#### Changes in skills

The technology use provided new skills, such as technical know-how. Professionals learnt how to deal with technology equipment in practice, e.g., set up alarms and use a variety of systems. Older people learnt new ways to use technology and receive help at home. Caregivers were also encouraged to use technology. Through received and analysed information, professionals gained insight and were able to provide better and safer care. Older people were able to cope at home independently and became aware of their capabilities, and caregivers got to know older people’s wellbeing at home. Technology use made professionals’ work more versatile, e.g., they used their guiding skills more and encountered older people in a new way. Older people understood new possibilities of technology use in safe care, and caregivers discovered new ways to be involved in the daily lives of older people. Moreover, professionals considered that technology use aids them in controlling their work, e.g., they were able to direct resources better than before and anticipate contacts. On the other hand, there were also responses suggesting that there were not enough experiences about technology use yet. Technology use was considered not to provide new skills to anyone, it did not involve older people or caregivers, or it was not in use. It was also said to provide false information, there were no caregivers around or they started to intrude in care too much, or the question was left blank, e.g., it was not answered.

#### Changes in information provision

Technology use provided information about older people’s wellbeing at home. Professionals described that they can holistically evaluate older people’s wellbeing and monitor changes in their health status. Older people received information that their wellbeing is followed up on and they can cope safely at home. When caregivers received information, they were able to track older people’s condition and safety. Technology use provided real-time information about realisation of care. Professionals were able to track medication intake and focus on gaps in care, for example. Older people were aware about daily practices in care, and caregivers received information about that, meaning that the older people were cared for in a timely way. Technology use provided knowledge about new possibilities in care. Professionals started to understand a variety of needs where technology use can be helpful. Older people received information about new ways to get in contact with others and to be active in society, and caregivers understood new ways to be involved in older people care. On the other hand, there were descriptions that technology was not in use, participants did not have enough experience yet, technology use did not provide any new information, but instead unnecessary and false information, information was not monitored, older people were told not to understand it, it was not known who had the devices, or the question was left blank.

#### Changes in procedures

Information provision was a new procedure that was formed with technology use. Professionals offered both practical help and discussions via technology with the older people, caregivers and each other. Older people received information about technology use, and caregivers were able to provide additional technology support to the older people and receive information about how they coped. New procedures for coping were developed at home. Professionals developed new processes to work, to deal with technology use, and to anticipate and react to the needs of older people. Older people combined ways to cope with technology use and became assisted by others, and caregivers had more options to contact the older people and the professionals. On the other hand, there were not enough experiences yet, technology use did not provide any new procedures, it brought extra work and unnecessary and false information, information was not monitored, there were no caregivers around, caregivers started to interfere in older people care, or the question was left blank.

#### Possibilities to do existing procedures differently

Visits and caring were able to be modified based on needs. Professionals received information easily and they were able to reconsider the urgency of the care they needed. Older people did not need to wait for the care, and caregivers no longer needed to be committed physically to older people care so much. Involvement was improved. Professionals were able to motivate the older people to be more active in care and in daily life. Older people were able to be active and be involved in care practice procedures, and older people and caregivers were able to contact each other easily from long distances. Nevertheless, there were not enough experiences yet, participants did not use technology, technology use did not offer differences to existing procedures, it did not involve older people or caregivers, or the question was left blank.

#### Existing procedures that can be left as being unnecessary

Some concrete procedures were able to be left out. Professionals did not need to travel to the older people’s homes so much anymore, to ensure the realisation of care practices, and offer physical company to the older people. Older people were not dependent on home care visits, and caregivers did not need to make on-site visits to the older people. Older people did not need to be cognitively oriented, e.g., treatment and localisation was possible even if the older person could not memorise the medication or location. From the caregivers’ perspective, ensuring older people’s wellbeing was no longer needed so much, e.g., tracking, making check-up visits and relying on what they said. However, there were not enough experiences yet, there were no procedures that could be left because of technology use where the technology use increases the number of treatments, technology use does not involve older people or caregivers, or there is a fear that medication know how will get worse.

#### Changes in motivation

**Fig. 3:**
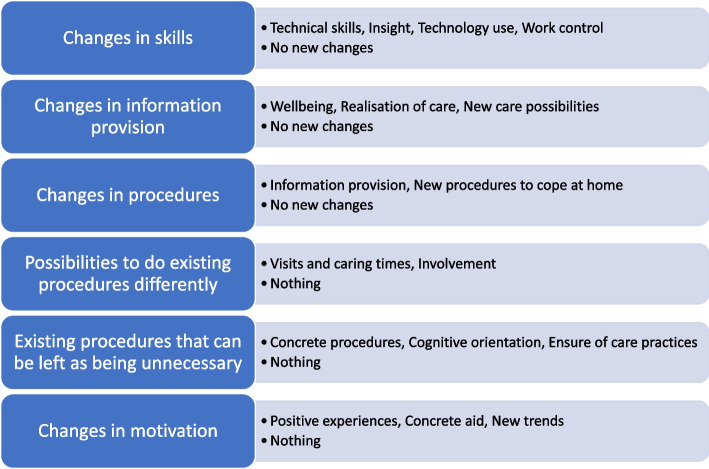
Topics and composed sub-categories that can lead to changes in home care

Technology use provided positive experiences. Professionals stated that as technology use was easy, they were pleased when the older people were satisfied with it. Older people were excited and got a feeling of success, and caregivers got excited when they noticed that the older people became inspired by technology use. Technology use provided concrete aid. Professionals’ work became easier, and they were able to allocate their resources more effectively. Older people were motivated when they had saw change in their independence and safety, and they saved money at the same time. Caregivers were motivated when older people were able to be independent, safe, and they saved their own efforts and money. New trends were also inspiring for professionals when they were able to develop new and rewarding ways to work. On the other hand, professionals described that nothing motivated them, the older people or the caregivers. They described that there was no time to get any of the advantages of technology use, it meant extra work and they were not interested in using it. They had gained no experiences yet from the older people or caregiver perspectives, and the use of technology originated from the professional, not the older people who might not even understand of technology use. The question was also left blank. Figure 3 describes the experiences.

### Experiences of how work and work processes change in technology use over time

After the technology was used for some time and the professional had gained experiences in its use, they started to see more new opportunities in care through the information it provides (10% vs 33%). They experienced technology use to be a new trend which motivates them (14% vs 37%). Previously, in the baseline survey they have considered that the most motivating factor in technology use is the concrete aid that it provides (62% vs 37%). From the older people’s point of view, follow-up shows that there was the greatest increase regarding insight in skills (43% vs 71%) and in information that the technology provides about wellbeing (25% vs 53%). Moreover, in the follow-up survey, respondents considered less than previously (45% vs 13%) that technology use does not provide new information (e.g., it was seen to provide more new information). From the caregiver point of view, the importance of concrete aid as a motivating factor was mentioned less (24% vs 0%) when technology had been in use. They also mention less (43% vs 23%) concrete procedures in the follow-up survey as procedures that can be left because they are unnecessary (e.g., they considered at the beginning that they do not need to make so many in-site visits to the older people anymore because visits become unnecessary, but at the follow-up survey they did not provide such opinions anymore). On the other hand, ensuring older people’s wellbeing was more necessary (24% vs 54%) while technology was in use. Changes can be seen in Table 3.

**Table 3:** Experiences of professionals of what topics and sub-categories may or may not lead to changes in technology use

Topics and composed sub-categories	Time points					
	Baseline (*N* = 21)^a^			Follow-up (*N* = 19)^a^		
	Professionals	Older people	Caregivers	Professionals	Older people	Caregivers
**Changes in skills**						
Technical skills	6/21 (29%)	3/21 (14%)	3/21 (14%)	4/19 (21%)	4/17 (24%)	1/19 (5%)
Insight^b^	6/21 (29%)	**9/21 (43%)**	10/21 (48%)	4/19 (21%)	**12/17 (71%)**	11/19 (58%)
Technology use	4/21 (19%)	8/21 (38%)	7/21 (33%)	5/19 (26%)	3/17 (18%)	6/19 (32%)
Work control	8/21 (38%)	N/A	N/A	7/19 (37%)	N/A	N/A
No new changes^b^	6/21 (29%)	**8/21 (38%)**	6/21 (29%)	3/19 (16%)	**2/17 (12%)**	5/19 (26%)
**Changes in information provision**						
Wellbeing^b^	**13/20 (65%)**	**5/20 (25%)**	8/20 (40%)	**8/18 (44%)**	**8/15 (53%)**	8/17 (47%)
Realisation of care	7/20 (35%)	3/20 (15%)	5/20 (25%)	5/18 (28%)	3/15 (20%)	4/17 (24%)
New possibilities in care^b^	**2/20 (10%)**	3/20 (15%)	2/20 (10%)	**6/18 (33%)**	4/15 (27%)	3/17 (18%)
No new changes^b^	5/20 (25%)	**9/20 (45%)**	6/20 (30%)	2/18 (11%)	**2/15 (13%)**	4/17 (24%)
**Changes in procedures**						
Information provision	7/21 (33%)	6/20 (30%)	5/21 (24%)	4/18 (22%)	3/15 (20%)	5/15 (33%)
New procedures to cope at home	17/21 (81%)	10/20 (50%)	8/21 (38%)	14/18 (78%)	8/15 (44%)	3/15 (20%)
No new changes^b^	**9/21 (43%)**	2/20 (10%)	10/21 (48%)	**4/18 (22%)**	4/15 (27%)	8/15 (44%)
**Possibilities to do existing procedures differently**						
Visits and caring times	16/21 (76%)	7/21 (33%)	8/21 (38%)	13/17 (76%)	5/15 (33%)	7/16 (44%)
Involvement^b^	4/21 (19%)	**6/21 (29%)**	5/21 (24%)	6/17 (35%)	**8/15 (53%)**	3/16 (19%)
Nothing^b^	5/21 (24%)	**9/21 (43%)**	10/21 (48%)	3/17 (18%)	**3/15 (20%)**	6/16 (38%)
**Existing procedures that can be left as being unnecessary**						
Concrete procedures^b^	17/21 (81%)	8/21 (38%)	**9/21 (43%)**	14/17 (82%)	8/15 (53%)	**3/13 (23%)**
Cognitive orientation	N/A	3/21 (14%)	N/A	N/A	4/15 (27%)	N/A
Ensure^b^	N/A	N/A	**5/21 (24%)**	N/A	N/A	**7/13 (54%)**
Nothing^b^	6/21 (29%)	**10/21 (48%)**	9/21 (43%)	6/17 (35%)	**3/15 (20%)**	5/13 (38%)
**Changes in motivation**						
Positive experiences^b^	9/21 (43%)	8/20 (40%)	**5/21 (24%)**	9/19 (47%)	6/17 (35%)	**0/15 (0%)**
Concrete aid^b^	**13/21 (62%)**	**12/20 (60%)**	16/21 (76%)	**7/19 (37%)**	**14/17 (82%)**	10/15 (67%)
New trends^b^	**3/21** **(14%)**	N/A	N/A	**7/19** **(37%)**	N/A	N/A
Nothing	5/21 (24%)	6/20 (30%)	3/21 (14%)	4/19 (21%)	2/17 (12%)	5/15 (33%)

^a^The number of team responses out of all responses. Professionals did not respond to all the topics from the perspective of older people or caregivers

^b^The biggest changes > 20 marked in bold

## Discussion

We studied how to adopt technology to meet the needs of professionals, older people and caregivers in home health care. We listed what technologies were piloted and deployed in a governmental programme, described the requirements for technology use when a variety of technologies were used in home care, and assessed the changes that take place in work and work processes when technology is adopted in home care.

There were 80 technology pilots, in which variety of technologies were piloted and deployed within nine months in the KATI projects. It took around one hour for the older people and 3.5 h for the professionals to learn to use the technology. There was huge variation between technologies, their maturation, sample sizes and ways to conduct the use: some technologies were tested in small pilots, but some were more widely used. In some projects, there were centralised personnel taking care of technology, while there were also projects in which personnel took care of technology maintenance during home health care.

From the professional, older people and caregiver perspectives, professionals considered familiarity, commitment, and understanding of technology benefits to be requirements for technology to be used. So far, the technology use had been positive, easy-going, successful, and it was thought that use would continue in the future. However, negative experiences were also mentioned, and a variety of different, even conflicting experiences were described by professionals, older people or caregivers. The received benefits are in line with previous studies in which usefulness [17, 19, 24, 26], and keeping older people up with the world [22] have been stated as being important in technology use. Ease of use [19, 27] and technology performance [19] have also been found to support the use in daily practice. Confidence in technology use to continue has previously also been said to depend on finances [13, 26]. As Mannheim et al. (2021) [21] have stated before, the attitudes of home care professionals can potentially affect how older adults are viewed in relation to technology, and it may either promote or hinder the adoption of technology. Therefore, it is also important to bring out gaps in technology use to meet the needs of users and to elucidate challenges in work processes, which were also recognised in this study.

Familiarity and commitment were described as important from the professional, older people and caregiver perspectives, whereas these factors have not received much attention in previous literature. Caregivers’ technology adoption has found to depend on their comprehension [16]. However, it may be evident that familiarisation is needed, and it may also be possible that commitment is considered as one form of motivation. Moreover, privacy [24–26], security [25] and reliability [26] have been stated before but were not highlighted from multiple perspectives in our study. According to Vandemeulebroucke et al. (2021) [14], ethical issues have rarely been described in studies of socially assistive robots, for example. One explanation may be that laws, privacy guidelines and network infrastructures have been set up to ensure that the procedures are secure and confident. According to the Privacy Shield Framework (2023), the Data Center Risk Index 2016 has graded Finland as the safest data centre location in the European Union and the fourth safest in the world [9]. Still, more effort is required to develop legal clarity, standardised documentation, and recommendations [12]. Moreover, socio-demographic variables did not receive any attention in our study. According to the existing literature, there is inconsistent and weak evidence on the impacts of socio-demographic variables and technology adoption [13].

From all our study perspectives, there were experiences at baseline of what may lead to changes in technology use. Technology use was thought to provide new skills such as insight, which was responded by more than one third (around 40 percent) of the teams. Similar number of teams (around 43 percent) considered that technology use provides information in a new way about older people’s wellbeing. More than half of the teams (around 56 percent) expected the technology use to develop new procedures to cope at home. Technology use could enable to modify visits and caring times by focusing on participant needs, which was considered almost half (around 49 percent) of the team responses. Further, more than half (around 54 percent) of the teams considered technology use to enable some concrete procedures to be left out as unnecessary. Two thirds (around 66 percent) of the teams expected technology use to motivate participants through the gained concrete aid. On the other hand, around one third of the team responses were pessimistic. They had negative expectations and considered that technology is providing nothing new or changing anything. Professionals’ experiences also changed during technology use, in terms of how it affects work and work processes. They started to see more new possibilities in care, so the most motivating factor in use was no longer the concrete aid it provides. From the point of view of older people, professionals considered the technology to provide older people with more insight, new wellbeing information, and opportunities to find and leave out unnecessary procedures in care. From the perspective of the caregiver, professionals considered the importance of concrete aid to change at the same time as wellbeing of older people was being ensured.

The results are in line with previous research. Timely treatment and quality of care [12, 23], and timely information about older people [20] can be increased by offering them a wide range of technology services. Technology use also has the potential to add report functionality and increase speed [20]. It is important to maintain the user’s curiosity [24] and promote their self-efficacy [25], as competency, motivation and perceived burden affect decisions about whether to adopt the technology or not [16]. Self-efficacy in particular has appeared to play a role in intention to use, as well as the actual use of technology [25]. Therefore, being mindful of driving forces that encourage use, learning and a sense of pride [23] are needed. Both supportive (helping with everyday activities) and empowering (obtaining physical or educational training to help maintain capabilities) aspects in technology adoption [41] are of importance.

However, insight and concrete changes in work and work processes, the possibility to leave out unnecessary procedures in care, and finding out new ways to engage in older people’s lives have not received much attention in previous research. For example, in Finland, the possibility for professionals to cease making home visits to older people might have stood out because Finland is a largely dispersed county with long travelling distances. Moreover, older people’s health status was not described to be a relevant theme for older people’s technology use, which is a similar finding to that in the study by van Houwelingen et al. (2018) [25], while at the same time being in contrast with other previous literature [48]. It is an important and even conflicting outcome of technology use that it was found that more procedures could be left out as being unnecessary from the point of view of older people, while there were not so many such procedures from the perspective of caregivers. However, from the caregiver point of view, professionals considered that the importance of ensuring older people’s wellbeing would increase. Caregivers found new and necessary procedures to engage in older people’s lives. Previously, post-intervention acceptance factors have been found to be more nuanced than pre-implementation factors, indicating that first-hand experience with technology enables the provision of a tangible, extensive and in-depth overview of technology acceptance [17].

Ways to adopt technologies in home care that benefit from use and meet the needs of professionals, older people and caregivers mean that technologies are involved in daily life. Technology use is a means to change clinical practice and develop in work and learn new ways of working and interacting with older people and caregivers. Caregivers have a significant role as a source of support and as technology use facilitators, especially if it improves their psychological wellbeing, by easing their caregiving burden and relieving some of their responsibility [17], but at the same time, they can disadvantage it by taking over older people’s technological tasks [25]. Technology use is a means to getting benefits such as saving time, costs, and better allocation of resources, especially among professionals who are geographically dispersed and spend a lot of their time in the field. It is also a means of being aware of older people’s safety and their ability to cope at home in distance-based care. Technology use means continuous on-site training, variation, and communication throughout an organisation. Its use provides opportunities, for example, for such professionals who are unable to work at the older people’s home due to physical restrictions. Therefore, projects designed new work practices and processes, integrated technologies into information systems and planned ways to fit them into ongoing practices, as technology itself does not make any changes. Moreover, they educated professionals as they recognised the need for a positive attitude and accomplished many things to support it. However, more is needed to prevent technology from being surrounded by negativity and so that colleagues no longer must encourage each other to use it [20].

### Strengths and limitations

One strength of the study is that mixed methods data provided insight into real-world home care regarding variety of technologies, and between intended and actual use. By using mixed methods design, we were able to merge qualitative and quantitative data, convert one type of data into the other type of data [36, 37] and discuss the results by comparing them. Wild et al. (2021) [49] have argued that most technology acceptance models focus on perceived usefulness and ease of use and equate the intent to use technology with actual use. Actually, there are multiple intervening variables suggesting that ethical, institutional and social factors need to be considered in use [49]. Another strength is that our study had a relatively large sample of participants. It was also carried out in several municipalities, with a variety of organisational structures.

There are also several study limitations. First, the KATI projects piloted and adopted a variety of technologies according to the needs of the regions with their varying scopes. Thus, there are challenges to achieving coherent results, even though the factors that affect technology adoption have been found not to differ across types of technologies [13]. Second, interview and team survey questions were not pilot-tested beforehand with the professionals. However, to increase the validity, results were discussed between researchers and verified by the research team. Results were also verified by home care professionals of the KATI programme by asking whether the preliminary analysis reflected their experiences. Overall, their experiences were very similar to the data, and reliability was considered to have been achieved when the data provided answers to the questions. Third, because of COVID-19, a shortage in personnel, and reform taking place in Finland, we had to focus on professionals’ experiences and collect only second-hand data from the older people and caregiver perspectives. Still, we are quite convinced that professionals are aware of the study topics from the various perspectives in their everyday working environments. Fourth, there were several simultaneous studies and nationwide questionnaires taking place in Finland. As professionals working at home do not have standard office hours, it was hard to reach them by email or they may have considered our requests as unimportant. Fifth, even though the technology categorisation was carried out to describe a variety of piloted technologies, the data was analysed as a whole. There were technologies that were just piloted, and it was found that it is not worth continuing as it does not work, and there were also technologies that were put into continuous use. Moreover, some of the technologies had several functionalities in them. Therefore, even though it is important that information about technology-specific data has been widely studied, there is still a need to gain experiences about all kinds of technologies in daily practice.

## Conclusions

In the future, we need to identify older people who may benefit the most from technology use and target the technology to the person’s interests and resources. Further research can also elucidate professionals’ specific roles and responsibilities in technology use, facilitate evidence-based competencies, contribute to personal and organisational readiness and maturity for technology adoption, and identify factors that help technology to become more widely adopted, even as supplementary care or individual use before home care is needed. The success of technology, including the level of adoption by users, is dependent on the end-users’ interaction with the technology, their skills and belief that the use of the technology will benefit their health and life, and on available funding and policy decisions.

### Supplementary Information

**Additional file 1:** **Supplementary file 1.** The interview guide modified from Technology Acceptance Model, Davis 1989, developed for the study. **Supplementary file 2.** The questionnaire modified from DirVA PROVE-IT, Lillrank et al. 2019, developed for the study.

## Data Availability

The datasets used and analysed during the current study are available from the corresponding author on reasonable request.

## Abbreviations

KATI: Smart Ageing and Care at Home
DirVA PROVE-IT: Prove Outcomes, Value, and Effectiveness of IT in Healthcare
THL: Finnish Institute for Health and Welfare
TAM: Technology Acceptance Model
