# Supplementary material for: How to adopt technologies in home care: a mixed methods study on user experiences and change of home care in Finland
Source: BMC Health Serv Res. 2023 Dec 2;23:1342. doi: 10.1186/s12913-023-10368-z (PMC10693073; doi:10.1186/s12913-023-10368-z)
Supplement: Supplementary file 1 — Additional file 1: Supplementary file 1. The interview guide modified from Technology Acceptance Model, Davis 1989, developed for the study. Supplementary file 2. The questionnaire modified from DirVA PROVE-IT, Lillrank et al. 2019, developed for the study. [file 12913_2023_10368_MOESM1_ESM.docx]

Supplementary file 1. The interview guide modified from Technology Acceptance Model, Davis 1989, developed for the study

1. What is required of the technology in use? From the point of view of

**professionals**

**older people**

**caregivers**

1. How does the use of technology contribute to everyday life? From the point of view of

**professionals**

**older people**

caregivers

1. What is the use of this technology like? From the point of view of

**professionals**

**older people**

caregivers

1. What is the attitude towards the use of technology? From the point of view of

**professionals**

**older people**

caregivers

1. How is the use of technology expected to continue? From the point of view of

**professionals**

**older people**

**caregivers**

Supplementary file 2. The questionnaire modified from DirVA PROVE-IT, Lillrank et al. 2019, developed for the study

- 1. What new skills does technology bring to personnel?

What new skills does technology bring to the older people?

What new skills does technology bring to caregivers?

- 1. What information does technology bring to personnel?

What information does technology bring to older people?

What information does technology bring to caregivers?

- 1. What new functions will be created for the personnel as a result of technology?

What new functions will older people have with the technology?

What new functions will be created for caregivers as a result of technology?

- 1. What previous activities can personnel do differently with technology?

What previous activities can older people do differently with technology?

What old activities can caregivers do differently with technology?

- 1. What functions will be redundant for personnel due to technology?

Which functions will be redundant for older people due to technology?

Which functions will become redundant for caregivers due to technology?

- 1. What motivates personnel to use technology?

What motivates older people to use technology?

What motivates caregivers to use technology?
